# TNF-α/TNFR1 activated astrocytes exacerbate depression-like behavior in CUMS mice

**DOI:** 10.1038/s41420-024-01987-4

**Published:** 2024-05-06

**Authors:** Mengjiao Gao, Yu Song, Yaqi Liu, Yuqing Miao, Yanwu Guo, Huihui Chai

**Affiliations:** 1 Neurosurgery Center, Department of Functional Neurosurgery, The National Key Clinical Specialty, Guangdong Provincial Key Laboratory on Brain Function Repair and Regeneration, The Neurosurgery Institute of Guangdong Province, Zhujiang Hospital, Southern Medical University, Guangzhou, 510282 Guangdong China; 2 Department of Cerebrovascular Surgery, The Third Affiliated Hospital of Sun Yat-Sen University, No 600 Tianhe Road, Guangzhou, 510630 Guangdong China; 3 Department of Neurosurgery, Huashan Hospital, Shanghai Medical College, Fudan University, National Center for Neurological Disorders, National Key Laboratory for Medical Neurobiology, Institutes of Brain Science, Shanghai Key Laboratory of Brain Function and Regeneration, Institute of Neurosurgery, MOE Frontiers Center for Brain Science, Shanghai, 200040 China

**Keywords:** Depression, Chronic inflammation

## Abstract

Neuroinflammation is considered to be a significant mechanism contributing to depression. Several studies have reported that A1 astrocytes were highly prevalent in human neuroinflammatory and neurodegenerative diseases. However, the precise mechanism by which A1 astrocytes contribute to depression remains unclear. Clinical studies have suggested a correlation between TNF-α, an activator of A1 astrocytes, and the severity of depression. Based on these findings, we hypothesized that TNF-α might worsen depression by activating A1 astrocytes. Our previous studies indicated that Rhodomyrtone (Rho) has the potential to improve depression-like behavior in mice. However, the exact mechanism for this effect has not been fully elucidated. Importantly, it was reported that Rho alleviated skin inflammation in a mouse model of psoriasis by inhibiting the expression of TNF-α. Based on this finding, we hypothesized that rhodomyrtone may exert antidepressant effects by modulating the TNF-α pathway. However, further research is required to investigate and validate these hypotheses, shedding light on the relationships between neuroinflammation, A1 astrocytes, TNF-α, and depression. By obtaining a deeper understanding of the underlying mechanisms, these findings could lead to the development of novel antidepressant strategies that target the TNF-α pathway in the context of neuroinflammation. In vivo, based on the established chronic unpredictable mild stress (CUMS) mouse depression model, we characterized the mechanism of TNF-α and Rho during depression by using several behavioral assays, adeno-associated virus(AAV) transfection, western blotting, immunofluorescence, and other experimental methods. In vitro, we characterized the effect of Rho on inflammation in TNF-α-treated primary astrocytes. TNFR1 expression was significantly increased in the hippocampus of depression-like mice, with increased astrocytes activation and neuronal apoptosis. These processes were further enhanced with increasing levels of TNF-α in the cerebrospinal fluid of mice. However, this process was attenuated by knockdown of TNFR1 and infliximab (Inf; a TNF-α antagonist). Injection of rhodomyrtone decreased the expressions of TNFR1 and TNF-α, resulting in significant improvements in mouse depression-like behaviors and reduction of astrocyte activation. TNF-α could be involved in the pathophysiological process of depression, through mediating astrocytes activation by binding to TNFR1. By blocking this pathway, Rho may be a novel antidepressant.

## Introduction

Depression is a multi-factorial psychiatric disorder characterized by a persistent depressed mood, loss of interest or pleasure, significant weight loss, and insomnia or hypersomnia, and is associated with a high risk of suicide and disability [1–3]. However, the specific pathogenesis of depression is still not well understood, so it is important to further understand the pathogenesis of this disorder. The antidepressants currently in clinical use act slowly, so patients can suffer from several side effects and resistance to drugs [4, 5]. In addition, approximately 50% of patients with major depression have problems with recurrent illness and relapse [6]. The development of faster-acting antidepressants is therefore important to improve adherence and reduce the harmful effects of delayed treatments.

Neuroinflammation, a common disease mechanism, has been found to play an important role in the pathophysiology of psychiatric disorders such as depression [7]. The major elements of the neuroinflammatory reaction include glial cells, the blood-brain barrier, inflammatory factors, and inflammatory signals [8]. Psychosocial stress, as one of the important trigger factors of mental disorders, can stimulate inflammatory signaling molecules [9]. In particular, the hippocampus plays an important role in depression [10]. Compared to non-depressed patients, depressed patients show an increase in relevant inflammatory cytokines such as tumor necrosis factor-alpha (TNF-α) and interleukin (IL) in peripheral blood and cerebrospinal fluid [11].

Initially, depression models focused on neurons and microglia [12, 13], and astrocytes have recently been found to become “active” under extreme conditions during inflammation, such as Alzheimer’s disease (AD), Parkinson’s disease, and multiple sclerosis (MS), undergoing dramatic changes in morphological, molecular, and functional levels [14–16]. Among them, type A1 astrocytes are usually induced by pro-inflammatory factors. TNF-α is a crucial factor that promotes conversion of astrocytes to type A1 astrocytes [17]. TNF-α has been found to simultaneously activate the receptors, TNFR1 and TNFR2 [18]. The binding of TNF-α to each of these two receptors can result in different biological effects. TNFR1 is mainly involved in the pro-inflammatory response [19]. The A1 astrocyte-specific marker is complement 3 (C3) [17], and activated astrocytes can further secrete chemokine C-X-C-subunit ligand 1 (CXCL1) [20]. However, the role of A1-type astrocytes activated by TNF-α in depression is currently unclear.

Clinical studies have suggested that inhibition of pro-inflammatory cytokines or their signaling pathways may improve depressed mood and increase responses to treatments with antidepressants [21].

Recently, Rhodomyrtone (Rho), a compound extracted from Rhodomyrtus tomentosa, was reported to reduce skin inflammation in a psoriasis-like mouse model by inhibiting TNF-α expression [22]. Our previous study reported that Rho reduced the severity of depression in depression-like behavior mice [23], but the exact mechanism is still not clear.

In this study, we hypothesized that TNF-α activated astrocytes to progress to the A1 type by binding to its pro-inflammatory specific receptor, TNFR1, to induce the development of depression-like behavior in chronic unpredictable mild stress (CUMS) mice, whereas Rho could “block” the process of this pathophysiology, by acting as an antidepressant.

## Results

### Chronic stress increases the expression of TNFR1 in hippocampal CA3 region

**Fig. 1:**
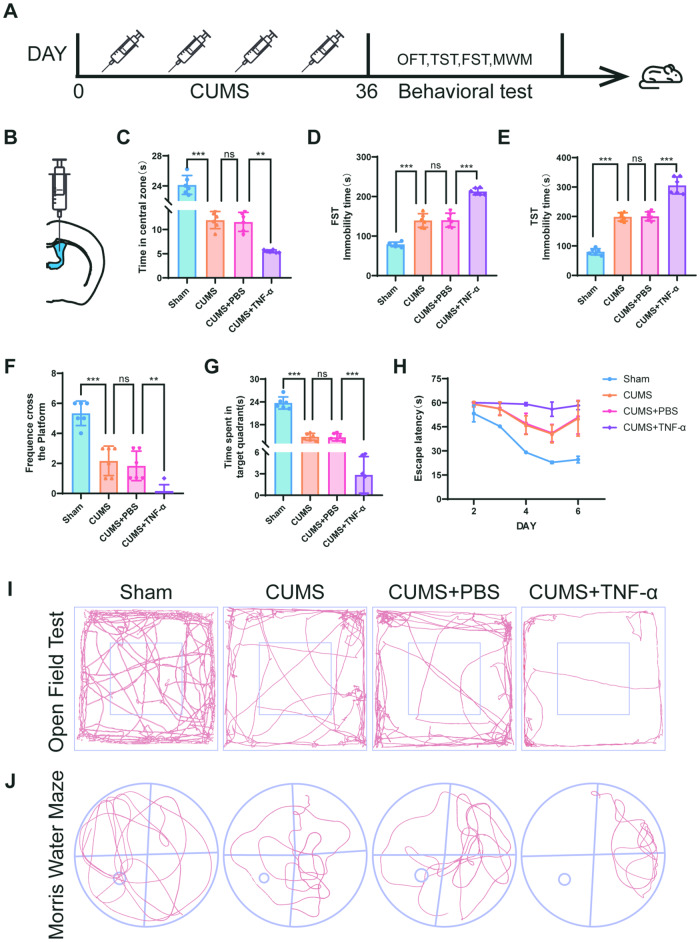
Depression-like behavior was examined with TNF-α treatment in the lateral ventricles of mice. **A** Schematic of the experimental design for assessing the effect of TNF-α, infliximab, and rhodomyrtone (injections four times every 7 days). **B** Schematic of the injection site in the lateral ventricle. **C** Chronic unpredictable mild stress (CUMS) mice spent less time in the center 25% area of the open field than the sham mice. TNF-α treatment aggravated the decrease of time in the center of the open field. **D** CUMS mice spent more immobility time in the last 4 min of the forced swimming test, when compared with sham mice. TNF-α treatment increased the immobility time in the CUMS group. **E** CUMS mice spent more immobility time in the tail suspension test than the sham mice. TNF-α treatment increased the immobility time in the CUMS group. **F** The number of platform crossings in each group. **G** Time spent in the target zone during the platform withdrawal period in the water maze. **H** Escape latency in all groups was improved with training. TNF-α treatment significantly aggravated escape latency in the CUMS group. **I** Typical tracks of open field exploration by one male mouse in each of the sham, CUMS, CUMS + PBS, and CUMS + TNF-α groups in exploration of the center areas. **J** Typical tracks of water maze exploration by one male mouse in each of the sham, CUMS, CUMS + PBS, and CUMS + TNF-α groups in exploration of each zone; *n* = 6 mice per group. Data are presented as the means ± SD. *P* values were analyzed using one-way analysis of variance. ^**^P < 0.01,^***^P < 0.001, ns no significance.

**Fig. 2:**
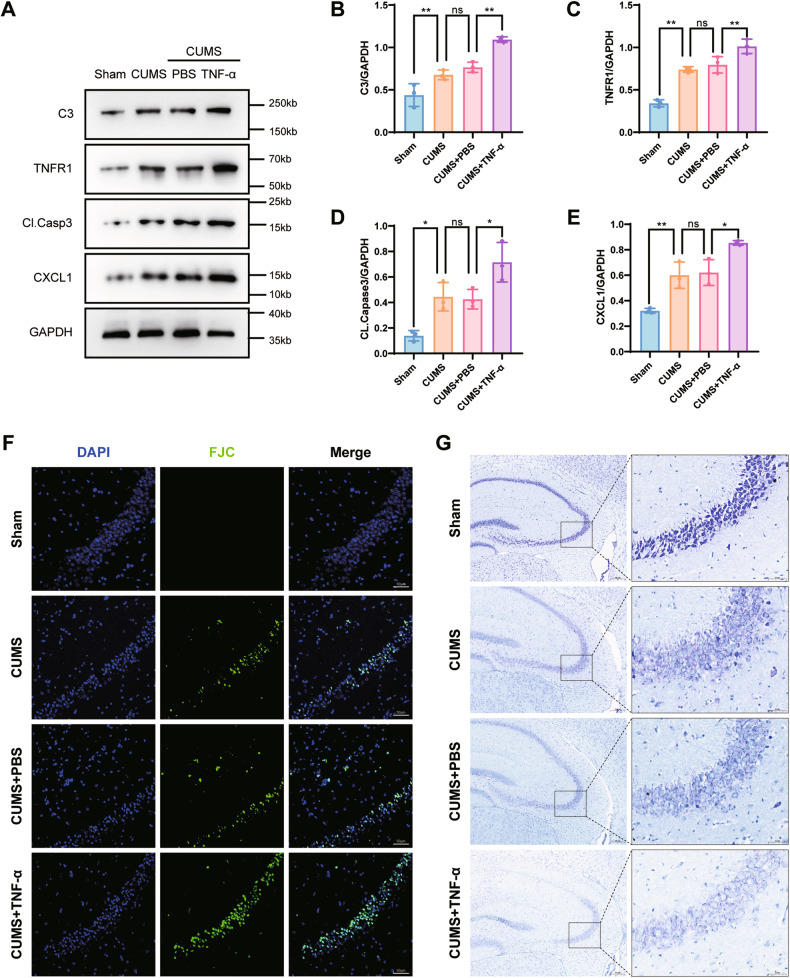
TNF-α aggravated astrocyte activation and neuronal apoptosis. **A** Complement 3, TNFR1, cleaved caspase3, and CXCL1 protein expressions in hippocampus of mice were analyzed in all groups. GAPDH was the loading control for the samples. TNF-α increased the activation of astrocytes and cleaved caspase3 expressions in chronic unpredictable mild stress hippocampus. **B**–**E** Quantitations of C3, TNFR1, cleaved caspase-3, and CXCL1 were normalized to GAPDH; *n* = 6 mice per group. **F** Representative images of FJC staining in the CA3 of hippocampus with TNF-α injection. Scale bar = 50 μm. **G** Representative image of Nissl staining in the CA3 of hippocampal brain tissue with TNF-α injection. Scale bar = 200 μm, scale bar of enlarged images = 50 μm. Western blot results were analyzed using ImageJ software. Data are presented as the means ± SD. *P* values were analyzed using one-way analysis of variance. ^*^P < 0.05,^**^P < 0.01, ns no significance.

Chronic stress is an important cause of depression, and long-term multiple and unpredictable mild stress can induce depression-like behaviors in mice. Compared with mice in the sham groups, chronically stressed mice showed significant depression-like behaviors using the open field test (OFT) (Figs. 1C and S3A), forced swimming test (FST) (Fig. 1D), and tail suspension test (TST) (Fig. 1E), and decreased learning and memory ability using the water maze test (Fig. 1F–H). The representative pictures of mouse movement trajectories in the OFT and MWM are shown in Fig. 1I, J. We used immunofluorescence and co-stained glial fibrillary acidic protein (GFAP) with TNFR1. The results showed that its expression in CA3 was higher than in the DG and CA1 regions of hippocampus in CUMS mice (Fig. S3B). The western blot results (Fig. S3C) showed that TNF-α protein expression was significantly increased in hippocampus in the CUMS mice. Immunofluorescence co-staining of TNFR1 and C3 (Fig. S3D); and co-staining GFAP with TNFR1 or C3 (Fig. S4), and western blot quantitation (Fig. 2A–E) showed that TNFR1, C3, cleaved caspase-3, and CXCL1 protein expressions were significantly increased in hippocampus in CUMS mice, when compared with the sham group. Fluoro-Jade C (FJC) (Fig. 2F) and Nissl (Fig. 2G) staining showed increased apoptosis of neurons in CUMS mice. These phenomena were more pronounced in CUMS + TNF-α mice when TNF-α levels in the cerebrospinal fluid were increased by stereotaxic injection. Taken together, these results showed that TNF-α overexpression promoted the development of depression.

### Inhibition of TNF-α expression in cerebrospinal fluid improves depression-like behaviors in mice

**Fig. 3:**
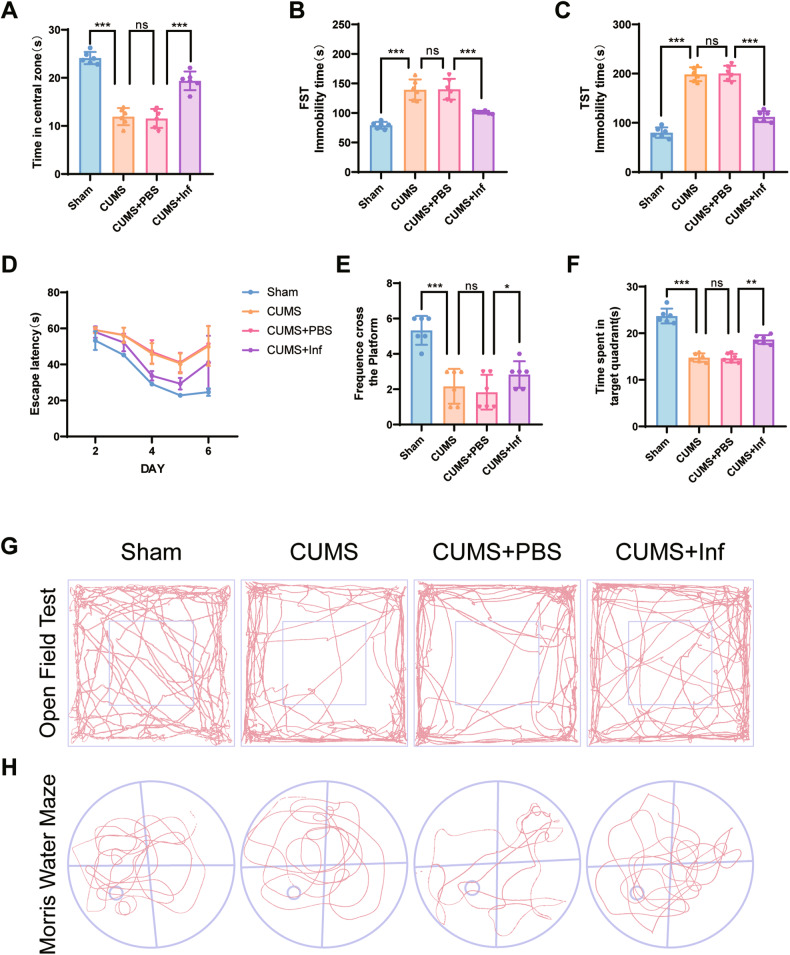
Depression-like behavior was examined with infliximab treatment in the lateral ventricles of mice. **A** Inf treatment alleviated the decrease of time in the 25% of the center area using the open field test. **B** Inf treatment reversed the increase of immobility time in the last 4 min of the forced swimming test in the chronic unpredictable mild stress (CUMS) group. **C** Inf treatment reversed the increase of immobility time in the tail suspension test in the CUMS group. **D** Escape latency in all groups was improved with training. Inf treatment significantly alleviated escape latency in the CUMS group. **E** The number of platform crossings in each group is shown. **F** Time spent in the target zone during the platform withdrawal period using the water maze test. **G** Typical tracks of open field exploration by one male mouse in each of the sham, CUMS, CUMS + PBS, and CUMS+Inf groups in exploration of the center areas. **H** Typical tracks of water maze exploration by one male mouse in each of the sham, CUMS, CUMS + PBS, and CUMS+Inf groups in exploration of each zone; *n* = 6 mice per group. Data are presented as the means ± SD. *P* values are analyzed using one-way analysis of variance. ^*^P < 0.05,^**^P < 0.01,^***^P < 0.001, ns no significance.

**Fig. 4:**
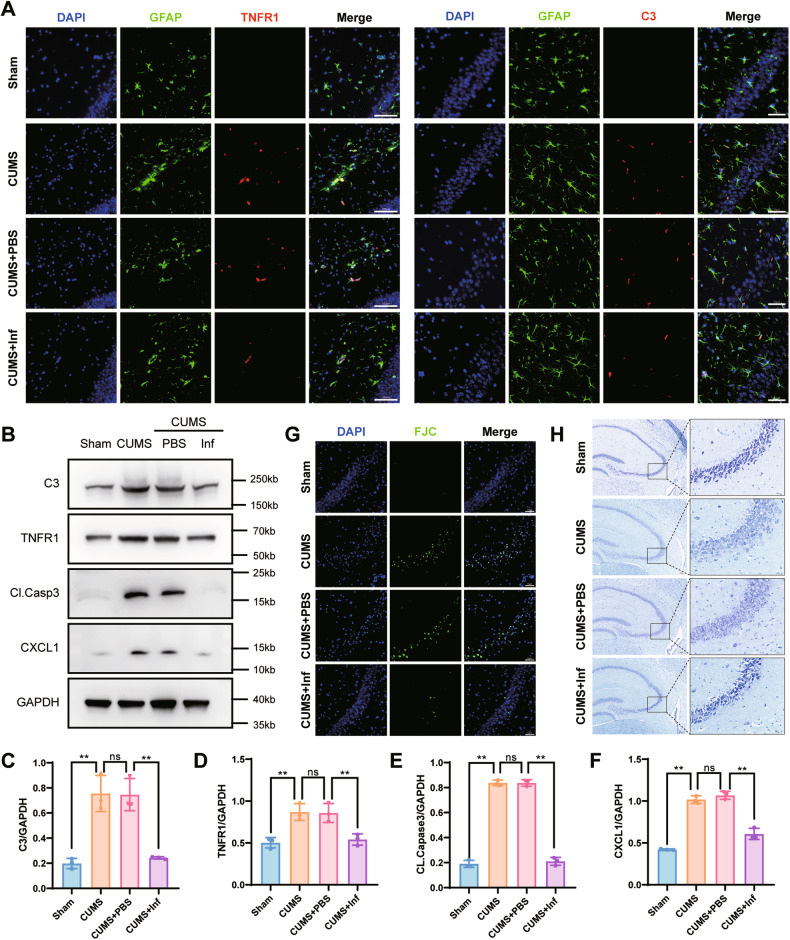
Infliximab (Inf) alleviated astrocyte activation and neuronal apoptosis. **A** Double fluorescence staining for GFAP (green) and TNFR1 (red) or C3 (red) in the CA3 of hippocampal with Inf treatment. Co-staining with GFAP and TNFR1, scale bar = 100 μm. Co-staining with GFAP and C3, scale bar = 50 μm. **B** Complement 3, TNFR1, cleaved caspase-3, and CXCL1 protein expressions in hippocampal brain tissue of mice were analyzed in all groups. GAPDH was the loading control for samples. Inf alleviated the activation of astrocytes and cleaved caspase-3 expressions in chronic unpredictable mild stress hippocampus. **C**–**F** Quantitations of C3, TNFR1, cleaved caspase-3, and CXCL1 were normalized to GAPDH; *n* = 6 mice per group. **G** Representative images of FJC staining in the CA3 of hippocampal with Inf treatment, scale bar = 50 μm. **H** Representative image of Nissl staining in the CA3 of hippocampus with Inf treatment, scale bar = 200 μm; scale bar of the enlarged image = 50 μm. Data are presented as the means ± SD. *P* values were analyzed using one-way analysis of variance. ^*^P < 0.05, ^**^P < 0.01, ns no significance.

To further demonstrate the role of TNF-α in depression, we injected infliximab (Inf), a TNF-neutralizing antibody [24], into the cerebrospinal fluid of mice. In behavioral tests, we found that Inf-treated mice showed significantly less depressive-like behavior using the OFT (Figs. 3A and S5A), FST (Fig. 3B), and TST (Fig. 3C), as well as significantly improved learning memory using the water maze test (Fig. 3D–F). The main results were an increase in exploration time in the 25% center of the open field and a decrease of immobility times using the FST and TST. The representative pictures of mouse movement trajectories in the OFT and MWM are shown in Fig. 3G, H. Immunofluorescence (Fig. 4A) and protein quantitation (Fig. 4B–F) results showed reduced TNFR1 and C3 protein levels in Inf-treated mice. In addition, FJC (Fig. 4G) and Nissl staining (Fig. 4H) showed that neuronal apoptosis was also significantly attenuated. Together, the results indicated that decreasing TNF levels improved depression-like behaviors in CUMS mice.

### CA3 knockdown of TNFR1 improves depression-like behavior

**Fig. 5:**
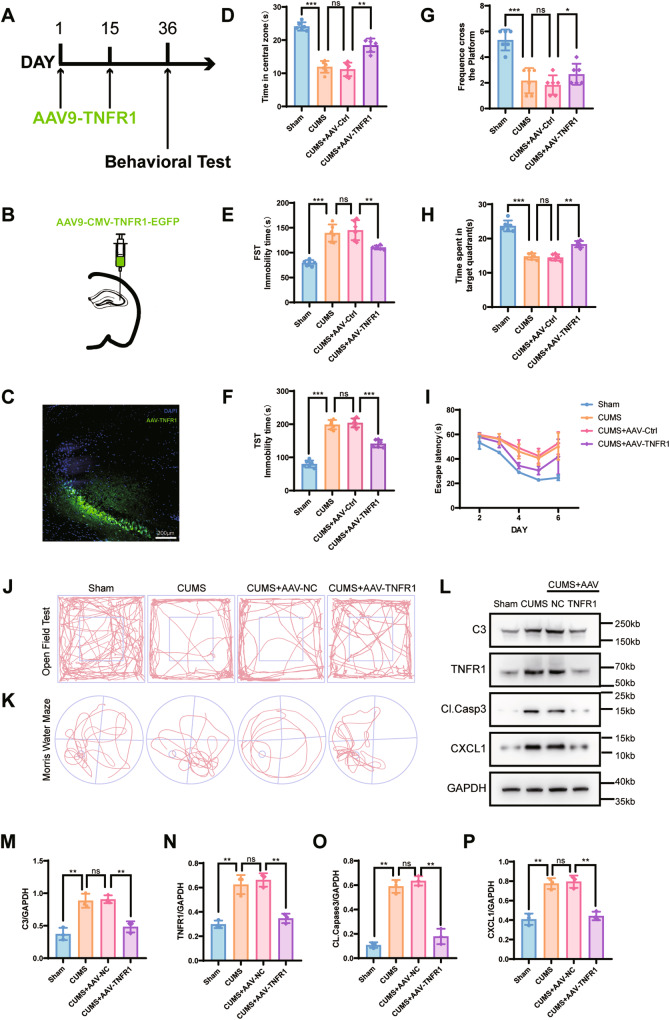
Depression-like behavior and protein expression were examined with AAV-TNFR1 transfection in hippocampus of mice. **A** Schematic of the experimental design for assessing the effect of AAV-TNFR1 transfection during in vivo experiments (injections two times every 15 days). **B** Schematic of the injection site in the CA3 of the hippocampal. **C** The expression of AAV-CMV-TNFR1-EGFP in the CA3, scale bar = 200 μm. **D** AAV-TNFR1 transfection alleviated the increase of time in the center 25% area of the open field in the chronic unpredictable mild stress (CUMS) group. **E** AAV-TNFR1 transfection reversed the increase of immobility time using the last 4 min of the forced swimming test in the CUMS group. **F** AAV-TNFR1 transfection reversed the increase of immobility time in the tail suspension test in the CUMS group. **G** The number of platform crossings in each group. **H** Time spent in the target zone during the platform withdrawal period in the water maze. **I** Escape latency in all groups was improved with training. AAV-TNFR1 transfection significantly alleviated escape latency in the CUMS group. **J** Typical tracks of open field exploration by one male mouse in each of the Sham, CUMS, CUMS + AAV-NC, and CUMS + AAV-TNFR1 groups in exploration of the center areas. **K** Typical tracks of water maze exploration by one male mouse in each of the Sham, CUMS, CUMS + AAV-NC, and CUMS + AAV-TNFR1 groups in exploration of each zone. **L** Complement 3, TNFR1, cleaved caspase-3, and CXCL1 protein expressions in the hippocampus of mice were analyzed in all groups. GAPDH was the loading control for samples. AAV-TNFR1 transfection alleviated the activation of astrocytes and cleaved caspase-3 expressions in the CUMS hippocampus. **M**–**P** Quantification of C3, TNFR1, cleaved caspase-3, and CXCL1 were normalized to GAPDH; n = 6 mice per group. Western blot results were analyzed using ImageJ. Data are presented as the means ± SD. *P* values were analyzed using one-way analysis of variance. ^**^P < 0.01, ^***^P < 0.001, ns no significance.

To determine whether treatment with TNF-α affected depression, considering the TNFR1 inducing pro-inflammatory effects, we constructed a GFP adeno-associated viral (AAV) vector carrying a knockdown TNFR1 gene driven by the CMV promoter (AAV-TNFR1; Fig. 5A, B). Using stereotaxic injection to bilaterally inject AAV-TNFR1 or AAV-NC in the CA3 region (Fig. 5C), the CA3 region of the experimental group was shown to have a large number of GFP-positive cells and reduced expression of TNFR1.

**Fig. 6:**
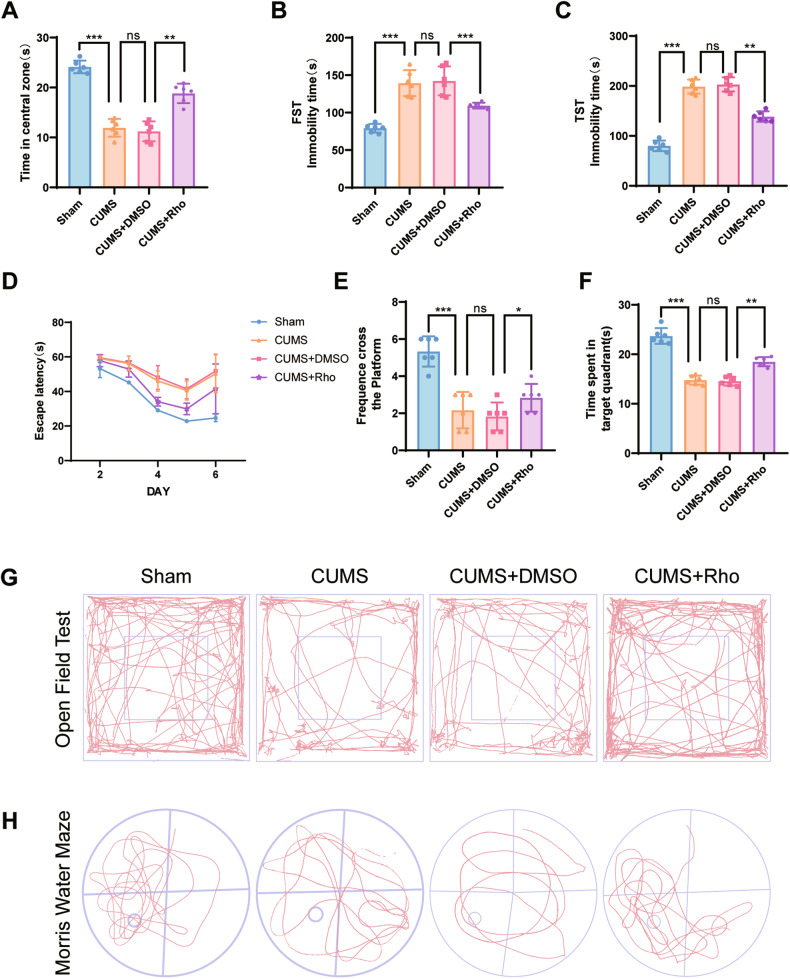
Depression-like behavior was examined with rhodomyrtone treatment in the lateral ventricles of mice. **A** Rho treatment alleviated the decrease of time in the center 25% area using the open field test. **B** Rho treatment reversed the increase of immobility time in the last 4 min using the forced swimming test in the chronic unpredictable mild stress (CUMS) group. **C** Rho treatment reversed the increase of immobility time in the tail suspension test in the CUMS group. **D** Escape latency in all groups was improved with training. Rho treatment significantly alleviated escape latency in the CUMS group. **E** The number of platform crossings in each group. **F** Time spent in the target zone during the platform withdrawal period in the water maze test. **G** Typical tracks of open field exploration by one male mouse in each of the sham, CUMS, CUMS + DMSO, and CUMS+Rho groups in exploration of the center areas. **H** Typical tracks of water maze exploration by one male mouse in each of the sham, CUMS, CUMS + DMSO, and CUMS+Rho groups in exploration of each zone; *n* = 6 mice per group. Data are presented as the means ± SD. *P* values were analyzed using one-way analysis of variance. ^*^P < 0.05, ^**^P < 0.01, ^***^P < 0.001, ns no significance.

AAV-TNFR1-transfected mice showed significant improvements in depression-like behaviors, when compared with mice in the AAV-NC and CUMS groups, such as a significant increase in the exploration time of the center of the open field (Figs. 6D and S5B) and a decrease of immobility time in the FST (Fig. 5E) and TST (Fig. 5F). In addition, the time to find the platform in the water maze (Fig. 6G–I) was shorter, with no significant degradation in learning memory ability. The representative pictures of mouse movement trajectories in the OFT and MWM are shown in Fig. 5J, K. Western blot quantitation (Fig. 5L–P) showed a significant reduction in protein expressions of C3 and CXCL1. Based on these results, knockdown of TNFR expression in mice induced antidepressive effects.

### Rhodomyrtone (Rho) mediates antidepressant-like effects through the regulation of TNF-α and TNFR1 binding pathways

Although our previous study confirmed the antidepressant effect of Rho, the exact mechanism was still unclear. We speculated that Rho may exert its antidepressant effect by inhibiting the binding of TNF-α to the receptor TNFR1. Based on this hypothesis, we injected Rho into the cerebrospinal fluid of mice.

**Fig. 7:**
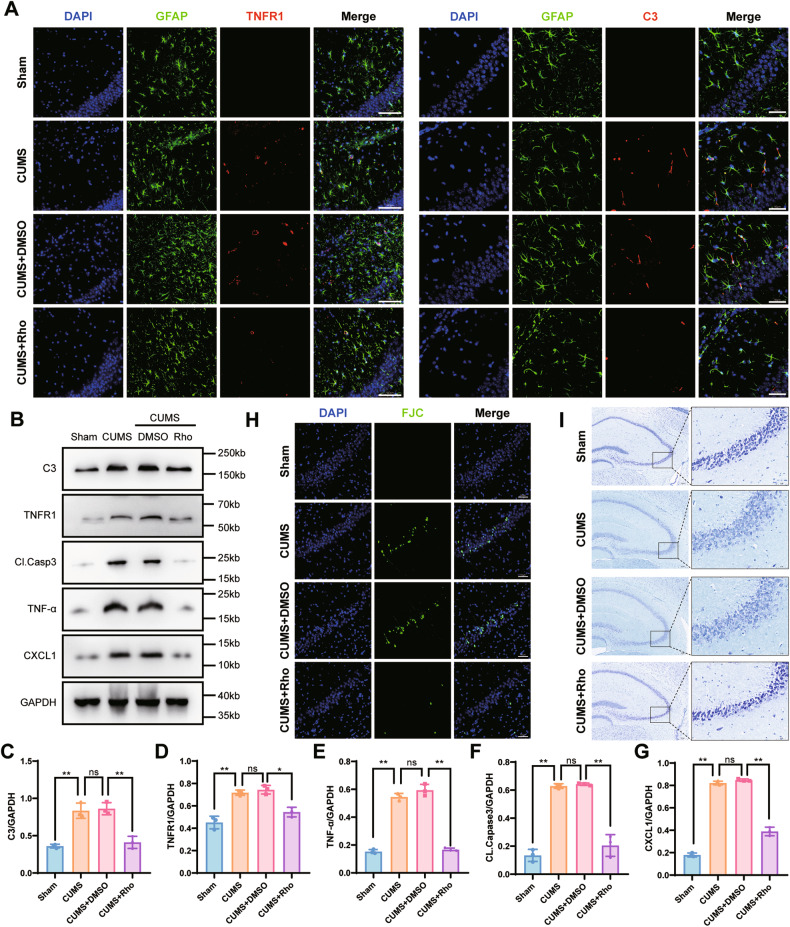
Rhodomyrtone alleviated astrocyte activation and neuronal apoptosis. **A** Double fluorescence staining for GFAP (green) and TNFR1 (red) or C3 (red) in the CA3 of hippocampal with Rho treatment. Co-staining with GFAP and TNFR1, scale bar = 100 μm; co-staining with GFAP and C3 scale, bar = 50 μm. **B** Complement 3, TNFR1, cleaved caspase-3, and CXCL1 protein expressions in hippocampal brain tissue of mice were analyzed in all groups. GAPDH was the loading control for samples. Rho alleviated the activation of astrocytes and cleaved caspase-3 expression in chronic unpredictable mild stress hippocampus. **C**–**G** Quantifications of C3, TNFR1, cleaved caspase-3, and CXCL1 were normalized to GAPDH; *n* = 6 mice per group. **H** Representative images of FJC staining in the CA3 of hippocampus with Rho treatment, scale bar = 50 μm. **I** Representative image of Nissl staining in the CA3 of hippocampal with rhodomyrtone treatment, scale bar = 200 μm; scale bar of enlarged image = 50 μm. Data are presented as the means ± SD. *P* values were analyzed using one-way analysis of variance. ^*^P < 0.05, ^**^P < 0.01, ns no significance.

In behavioral tests, Rho-injected mice showed significant improvements in depression-like behaviors using the open field test (Figs. 6A and S5C), FST (Fig. 6B), TST (Fig. 6C), and shorter platform finding time in the water maze test (Fig. 6D–F). The representative pictures of mouse movement trajectories in the OFT and MWM are shown in Fig. 5G, H. Immunofluorescence (Fig. 7A) and western blotting (Fig. 7B–H) quantitation showed that TNF-α, TNFR1, C3, and CXCL1 protein expressions were significantly decreased. Neuronal apoptosis was not obvious after FJC (Fig. 7G) and Nissl (Fig. 7H) staining. Overall, the results showed that Rho induced antidepressant effects by inhibiting TNF-α binding to TNFR1, resulting in astrocyte activation.

### Rho inhibits TNF-α-induced activation of astrocytes

To further investigate the mechanism of rhodomyrtone-induced antidepressant effects, using in vitro experiments, we extracted primary astrocytes and incubated them with TNF-α to stimulate their activation.

**Fig. 8:**
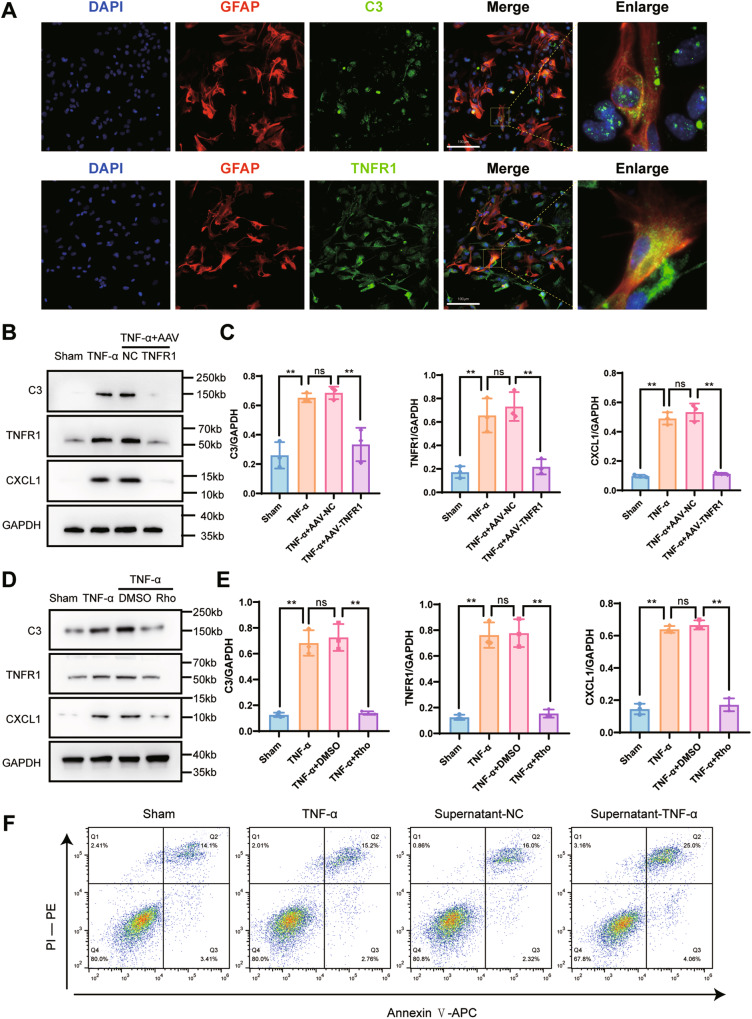
Rhodomyrtone or AAV-TNFR1 transfection alleviated astrocyte activation and neuronal apoptosis in vitro. **A** The expressions of TNFR1 and C3 in primary astrocytes. Double fluorescence staining for GFAP (red) and TNFR1 (green) or C3 (green) in the primary astrocytes with TNF-α treatment, scale bar = 100 μm. **B** Complement 3, TNFR1, cleaved caspase-3, and CXCL1 protein expressions of primary astrocytes were analyzed in all groups. GAPDH was the loading control for samples. AAV-TNFR1 transfection alleviated the activation of astrocytes with TNF-α treatment. **C** Quantitations of C3, TNFR1, and CXCL1 were normalized to GAPDH; *n* = 3 per group. **D** Complement 3, TNFR1, cleaved caspase-3, and CXCL1 protein expressions of primary astrocytes were analyzed in all groups. GAPDH was the loading control. Rho alleviated the activation of astrocytes with TNF-α treatment. **E** Quantitations of C3, TNFR1, and CXCL1 were normalized to GAPDH; *n* = 3 per group. **F** Effect of primary astrocytes supernatant with TNF-α treatment on HT22 cells. Representative dot plots showing the gating strategy of Annexin V-APC, and propidium iodide from different HT22 cell groups. Data are presented as the means ± SD. *P* values were analyzed using one-way analysis of variance. *P < 0.05, **P < 0.01, ns no significance.

We identified the primary astrocytes using co-staining with GFAP and IBA-1 (Fig. S5D). Cytotoxicity of TNF-α and Rho to astrocytes was then detected using a calcein AM/propidium iodide (PI) double staining assay, followed by flow cytometry. The results showed that TNF-α at 30 ng/mL or Rho at 30 μg/mL had no significant effect on astrocyte viability (Fig. S5E). We used immunofluorescence to localize the expression of TNFR1/C3 of astrocytes after TNF-α treatment (Fig. 8A). TNFR1 knockdown (Fig. 8B, C) and cultured astrocytes with Rho (Fig. 8D, E) both showed decreased protein levels of TNFR1, C3, and CXCL1. To detect the effect of activated astrocytes on neurons, we cultured HT22 cells with the supernatant of TNF-α-treated astrocytes, then determined the activity of HT22 cells using flow cytometry. The results showed that activated astrocytes induced neuronal apoptosis (Fig. 8F). Together, these results indicated that Rho inhibited TNF-α-induced activation of astrocytes.

## Discussion

In this study, we found that the adverse effects of TNF-α promoting stress-induced depression-like behavior in CUMS mice may involve binding to the TNFR1 to activate astrocytes. To further identify the mechanism of TNF-α during depression, we used CUMS mice as our in vivo experimental model. The results showed that chronic stress-induced depression-like symptoms in mice. Overexpression of TNF-α promoted depression-like behaviors in mice, but inhibition of TNF-α expression, knockdown of the proinflammatory receptor, TNFR1 of TNF-α, and injection of Rho attenuated the depression-like behaviors of mice. We found that Rho inhibited the protein expressions of TNF-α and TNFR1. Using in vitro experiments, we characterized the role of TNF-α on astrocytes, which showed that TNF-α activated astrocytes to the A1 type. Taken together, we hypothesized that TNF-α promoted depression-like behavior in CUMS mice by binding to TNFR1 to activate astrocytes, whereas Rho alleviated this process and thus exerted antidepressant effects.

Depression, a common stress-induced psychiatric disorder, has been closely associated with neuronal apoptosis and neuroinflammation, of which glia and cytokines play important roles [25–27]. Our results showed that chronic stress, inducing depression-like behaviors in mice, led to increased levels of TNF-α, TNFR1, C3, CXCL1, and cleaved-caspase-3, indicating that astrocyte activation and neuronal apoptosis occurred during these depression-like behaviors. Our results were consistent with several clinical studies that reported that levels of TNF-α were increased in peripheral and central neuronal systems of depressed patients, and was associated with the severity of depression (references). Our study may therefore provide additional explanations of how TNF-α exacerbates depression, suggesting that regulating levels of TNF may decrease depression.

The present study found that TNFR1, different from TNFR2, bound TNF-α, to mediate pro-inflammatory responses, such as activating immune cells to interact with each other and neurons, to promote degeneration of neurons [28, 29]. We also found that TNFR1 levels were upregulated in CUMS mice, whereas knockdown of TNFR1 levels improved the depression-like behavior in mice. Our results showed that alleviating inflammation resulting from knockdown of TNFR1 may have been the reason for the increase of neuron survival. Our study showed that inflammation plays an important role in the pathophysiology of depression, and suggests that antagonizing or blocking TNFR1 could be a target for antidepressant therapy.

Recently, it has been reported that astrocytes were initiated and modulated during inflammation in the central nervous system [30–32]. One study classified mouse astrocytes into two different states of activation: A1 and A2 [17]. They also showed that A1 astrocytes, activated by TNF-α, IL-1, and C1q secreted by activated microglia [17], which were abundant in neurodegenerative diseases [33], were neurotoxic and could cause neuronal death. C3-positive A1 astrocytes are present in most major human neurodegeneration diseases that involve neurodegeneration, such as Alzheimer’s disease, Huntington’s disease, Parkinson’s disease, and multiple sclerosis [34–36]. Thus, we assumed that A1 astrocytes were activated by TNF-α during depression. Our results indicated that the levels of C3, TNF-α, and TNFR1 were increased in the CUMS mice, and TNF-α could activate primary astrocytes, suggesting that TNF-α may activate pro-inflammatory astrocytes by TNFR1 signaling pathways during depression.

Clinical studies have reported that anti-inflammation improved depression, with the main trials currently focused on non-steroidal anti-inflammatory drugs (NSAIDs), and cytokine inhibitors [37–39]. However, NSAIDs do not improve depression [38]. In contrast, cytokine antagonists appear to be more promising. Infliximab only benefits treatment-resistant major depressive disorder (MDD) patients with elevated levels of inflammation [40]. Our previous study reported that Rho improved the depression-like behavior in mice [23].

Rhodomyrtone, a compound derived from Rhodomyrtus tomentosa, has significant antibacterial and anti-inflammatory effects [41, 42]. Chorachoo et al.(2018) reported that a low dosage of Rho inhibited the TNF-α-induced keratinocyte inflammatory response [43]. We therefore hypothesized that Rho induced an antidepressive effect by inhibiting the TNF-α pathway. Our results showed that Rho inhibited activation of A1 astrocytes by decreasing the levels of TNF-α and TNFR1, which is an important receptor in the activation of astrocytes. Rho also alleviated neuronal apoptosis and reversed the impairment of spatial memory in CUMS mice. Users of antidepressive agents currently experience several adverse effects, such as weight gain, sexual dysfunction, and treatment resistance [4, 44, 45]. Studies have shown that Rho had the advantages of low drug resistance and toxicity [46, 47], so Rho may be a promising antidepressant.

Our study had several limitations. For example, it did not use conditional knockout of TNFR1 in astrocytes in mice. Additionally, the mechanism of Rho involved in the regulation of depression needs to be further studied. Although Rho showed promising antidepressant effects, the intracerebral injection used in our study to avoid erythrocyte hemolysis caused by high concentrations of drug was not conducive to clinical translation.

In conclusion, our data provided new evidence that TNF-α/TNFR1 are crucial players in the process of depression. First, TNF-α could activate astrocytes to modulate neuroinflammatory responses in depression and promote neuronal degeneration by binding to TNFR1. Second, knockdown of TNFR1 could improve depression-like behavior in mice and inhibit activation of astrocytes. Finally, Rho alleviated stress-induced depression-like behavior and impairment of learning memory skills in mice. Furthermore, we showed that Rho may alleviate depression by decreasing the expressions of TNF-α and TNFR1. Taken together, our results suggest that Rho could be a promising treatment for depression.

## Methods

### Animals

The animals used in this experiment were 6–8-week-old C57BL/6 male mice purchased from the Animal Center of Southern Medical University. All experimental operations and animal husbandry were approved by the Ethics Committee of Southern Medical University (LAEC-2021-049FS2) and conformed to international animal health guidelines. The animals were housed in the animal center of Zhujiang Hospital of Southern Medical University with constant light, temperature, and humidity at the specific pathogen-free (SPF) level and acclimatized to the environment for 1 week before the experiments were conducted.

### Chronic unpredictable mild stress model

The CUMS mouse model chosen for this experiment was constructed based on previous studies with minor modifications [48–51]. The stresses involved in the experiment included water deprivation for 24 h, food deprivation for 24 h, a modified light/dark cycle for 24 h, soiled bedding for 24 h, cold water bath for 15 min (4 °C), restraint stress for 2 h, placement into a tilted cage 24 h per time (45°), stroboscopic light flashes 12 h per time, and tail pinches 5 min. The above stressors should be executed randomly for 36 days, and the stimulation interval of the same stressor should be greater than 5 days to avoid adaptation of experimental animals. Animals of sham groups were housed in the same environment but no stressors were added.

Before conducting the CUMS model, the open-field test was performed on the mouse. We excluded the insensitive subgroup by calculating the ratio (the time mouse stayed in the central area(25%) of the open filed experimental apparatus).

### Additional methods

See the Supplementary Methods and Materials for details of the remaining experimental design and methods.

The behavioral experiment video-tracked device was a Sony HDR-CX270 (Sony, NY, NY, USA), and the analysis software was EthoVision XT15 (Noldus, Beijing, China).

### Supplementary information

supplemental material

supplemental figure 1

supplemental figure 2

supplemental figure 3

supplemental figure 4

supplemental figure 5

Supplementary Table 1

Supplementary Table 2

Supplementary Table 3

blots

## Data Availability

The datasets used and analyzed during the current study are available within the manuscript and its additional files.
