# Supplementary material for: TNF-α/TNFR1 activated astrocytes exacerbate depression-like behavior in CUMS mice
Source: Cell Death Discov. 2024 May 6;10:220. doi: 10.1038/s41420-024-01987-4 (PMC11074147; doi:10.1038/s41420-024-01987-4)
Supplement: Supplementary file 1 — supplemental material [file 41420_2024_1987_MOESM1_ESM.docx]

**Supplemental methods and materials**

**Experimental Design and Groups**

The experimental design and drug dosage selection are as follows ( Fig.S1; S2):

**In vivo experiments** 126 6-8 weeks male C57BL/6 were randomly divided into nine groups ( 14 mice per group): Sham, CUMS, CUMS + PBS, CUMS + TNF-α (300ng/μl), CUMS + infliximab (300μg/μl), CUMS + DMSO(10%), CUMS + Rhodomyrtone (150μg/μl), CUMS + AAV-NC, CUMS + AAV-TNFR1. The CUMS + vehicle groups underwent the same brain stereotactic surgical procedure with injected the same volume of vehicle.

Behavioral tests (n=6 mice per group) (the open field test, forced swimming test, tail suspension test, Morris water maze) were used to determine degree of depressive-like behavior ,cognitive and learning ability in mice. The different behavioral tests should be carried out at an interval of more than 1 day. Behavioral teats were evaluated blindly that the surgical intercention on the mouses were blind to operator of the behavioral tests

Animals were sacrificed for brain or hippocampus tissue after behavioral tests. The samples were collected for immunofluorescence staining (n=3 mice per group), western blot (n=3 mice per group), fluoro-Jade C (FJC) (n=3 mice per group) and Nissl staining (n=3 mice per group). Western blot was used to determined the expression of GFAP, C3, TNFR1, cleaved caspased-3 and CXCL1. Double-labeled immunofluorescence was used for cellular localization of GFAP, C3 and TNFR1 in hippocampus. To detected neuronal apoptosis and degenerated, tunel staining and Fluoro-Jade C (FJC) staining was performed.

**In vitro experiments** Primary astrocytes were extracted from the cortex of newborn mice (less than 24h old, total n=120 mice). The vitro experiments included 6 groups: sham, TNF-α (30ng/ml), TNF-α + AAV-NC (3μl), TNF-α + AAV-TNFR1 (3μl), TNF-α + DMSO (10%), TNF-α + Rho (30μg/ml). Double-labeled immunofluorescence was used for cellular localization of GFAP, C3 and TNFR1. Western blot was used to determined the expression of C3, TNFR1 and CXCL1. To detected neuronal apoptosis and degenerated, tunel staining and Fluoro-Jade C (FJC) staining was performed. Flow cytometry assary was uesed to detected the apoptosis of neurons cultured with supernatant of astrocytes from different groups (sham, 30ng/ml TNF-α, supernatant from sham astorcytes, supernatant from astrocytes cultured with 30ng/ml TNF-α).

**Behavioral experiments**

**Open Field Test (OFT)**

Mice were placed in the behavioral room in advance for 30 min before the start of the experiment to adapt to the environment. OFTs were performed in the apparatus with a height of 50 cm, length of 50 cm, and width of 50 cm. A camera recorded the movements of mice within 5 min. The trajectory of mice and the time spent in the 25% center of the device were analyzed using EthoVision XT 15 software (Noldus, Beijing, China). At each test, the floor surfaces and walls of the apparatus were cleaned with 75% ethanol to abolish sign of olfactory cues.

**Forced Swimming Test (FST)**

A mouse was placed in a device with a height of 30 cm and diameter of 10 cm, which was filled with water at a temperature of 22 ± 2°C after acclimatizing to the environment for 30 min. The height of the water was such that the mouse’s hind limbs and tail could not touch the bottom of the device. The mouse’s behavior was recorded within 6 min using a camera, and the immobility time of the mouse in the last 4 min was analyzed. Immobility time was defined as the time the mouse did not make any movements other than those necessary to keep its nose above the water’s surface.

**Tail Suspension Test (TST)**

The mouse was suspended in a device with a height of 30 cm, width of 20 cm, and length of 20 cm by taping the tail after adapting to the environment for 30 min. Mice could not reach the apparatus wall with their limbs. The mouse’s behavior was recorded within 6 min using a camera, and the immobility time of each mouse was analyzed. Immobility time included minimal movements such as only forelimbs without hind leg movements, and pendulum-like movements.

**Morris water maze**

A circular pool of 120 cm in diameter was filled with an opaque white liquid, divided equally into four quadrants, with a 5 cm diameter platform in the center of one quadrant. During the first 5 days of training, the platform was approximately 1 cm below the water surface, and mouse was placed in the pool from four different positions, with each cycle lasting 60 s. On day 6, the platform was removed, and the mouse was placed in the pool from a fixed position. A camera recorded the movements of the mouse. Software was used to analyze the time it took for the mouse to find the platform each day, as well as the time it spent in the target quadrant, and the number of times it crossed over the platform after the platform period was removed. The mouse entered the water points and the platform locations as listed in Supplemental Table 1.

**Stereotactic brain injection**

The brain stereotactic injection areas in this experiment included the lateral ventricle and hippocampus. The mouse was fixed to the stereotaxic device through the ear bars and incisor holes after being anesthetized with tribromoethanol. Using the fontanelle on the skull surface as the zero point coordinate, the lateral ventricle coordinate axis for this experiment was -0.58 mm anteroposterior (AP), -1 mm mediolateral (ML), and -2 mm dorsoventral (DV), and the hippocampal coordinate axis was -1.82 mm (AP), -2 mm (ML), and -2 mm (DV). A total of 3 μL of reagent (TNF-α; Peprotech,, CA, USA; Infliximab, Abmole, TX, USA; Rhodomyrtone, YuanYe, Shanghai, China); or AAV(OBio, Shanghai, China) was injected at a rate of 1 μL/min.

In this experiment, We injected TNF-α, infliximab and Rhodomyrtone 1 time every week for 4 weeks. AAV was injected 2 times in 36 days with the interval of 15 days. We used the original AAV titers provided by biological company without diluted. AAV9-CMV-TNFR1-EGFP primers sequences used in this study were shown in Supplemental Table 2.

**Primary astrocyte extraction and culture**

Primary astrocytes were extracted from the cortex of newborn mice (less than 24 h old). The cortical tissue stripped of brain membranes was digested with 2.5% trypsin (Gibco, Grand Island, NY, USA) into individual cells, then digestion was terminated with complete medium (DMEM and penicillin-streptomycin solution, Gibco) with 10% fetal bovine serum (Gibco). The precipitated cells after centrifugation (300 × g; 5 min) were resuspended with complete medium, then incubated in an incubator (37°C; 5% CO2). The cells were identified as astrocytes by immunofluorescent labeling with GFAP and Iba-1( Fig. S2G).

**HT22 cells culture**

HT22 cells (CL-0687, Pricella, Wuhan, China) were cultured with complete medium (DMEM and penicillin-streptomycin solution, Gibco) with 10% fetal bovine serum (Gibco). Cells were passaged with 2.5% trypsin (Gibco) and incubated in an incubator (37°C; 5% CO2).

**Immunofluorescence staining**

After being anesthetized with tribromoethanol, mice were sequentially perfused with cold saline and 4% polyformaldehyde. The brain tissue was immediately removed and immersed in 4% paraformaldehyde for 24 h at 4°C, then dehydrated using 30% sucrose solution. The brain was embedded in OCT gel (Sakure, CA, USA)and snap-frozen, then sectioned to a thickness of 10 μm using a freezing microtome.

After fixing in methanol for 20 min, tissue sections were immersed in antigen repair solution for 10 min in a microwave oven and cooled to room temperature. After blocking for 1 h at room temperature using 10% donkey serum, the primary antibodies were incubated overnight at 4°C The primary antibodies were to TNFR1 (PA5-120358, 1:500; 16-1202-81, 1:500; Thermo Fisher Scientific, Waltham, MA, USA); C3: (PA5-114921,1:500; Thermo Fisher Scientific), and GFAP (14-9892-82, 1:500, Thermo Fisher Scientific). The secondary antibodies (14-9892-82, 1:500; A32766, 1:500; A-21110, 1:500; Thermo Fisher Scientific) were incubated at room temperature for 1 h and then stained with 4´,6-diamidino-2-phenylindole (DAPI) for 5 min. Images were obtained and analyzed under a fluorescence microscope (Nikon, TI2-E, Japan).

**Real-time quantitative PCR (qPCR)**

Total RNA was isolated from the primary astrocytes using TRIzol^TM^ Reagent (Takara , Beijing, China) according to the manufacturer’s instructions. RNA contents and the purity were measured by the ratio of absorbance at 260/280 nm and 260/230 nm. The primer sequences were found from website of PrimerBank and shown in Supplemental Table 3. The Evo M-MLV Reverse Transcription Kit (AG11728, agbio, Hunan, China) used to synthesize cDNA by SimpliAmp^TM^ Thermal Cycler (Thermo Fisher). The SYBR Green Pro Taq HS premixed qPCR kit (AG) was used for qPCR reactions by QuantStudio3 (Thermo Fisher).

RNA was normalization was based on the GAPDH gene expression. All data were obtained from at least three independent experiments and analyzed using QuantStudio^TM^ Design & Ananlysis Software.

**Western blotting**

Mice anesthetized with tribromoethanol were perfused with cold saline. After collecting hippocampal brain tissue, RIPA lysis buffer was added and the tissue was ground in a tissue grinder (70 Hz, 90 s, -30°C). The supernatant was collected after centrifugation (14,000 rpm, 15 min, 4°C) and heated for 10 min (100°C) by adding loading buffer. The 6% and 12% SDS-PAGE gels were prepared, followed by addition of 40 μg of protein sample per well of the 6% gel. Protein electrophoresis procedures involved 80 V for 30 min, and 120 V for 1 h. Transfer of protein to PVDF membrane used a constant current of 200 mA. After blocking for 1 h at room temperature using 5% nonfat milk, the membrane was incubated overnight at 4°C with primary antibodies against TNFR1 (PA5-120358, 1:1,000; Thermo Fisher Scientific); C3 (PA5-114921, 1:1,000; Thermo Fisher Scientific); CXCL1 (ab86436, 1:1,000; Abcam, MA, USA); cleaved caspase3 (9664, 1:1,000; CST, Mass, USA), and GAPDH (5174, 1:1,000; CST). After the membrane was incubated for 1 h at room temperature with secondary antibody (31460, 1:10,000; Thermo Fisher Scientific), the bands were identified using an ECL (NCM Biotech, Suzhou, China) chemiluminescence instrument and analyzed with the ImageJ software (ImageJ, National Institutes of Health, Bethesda, MD, United States). GAPDH was used as the internal control.

**FJC staining**

FJC staining was performed on brain tissue sections from different intervention groups using the FJC kit (Solarbio, Beijing, China) to detect neuronal degeneration. After hydration in distilled water for 3 min, frozen brain sections were stained in working solution A for 5 min, then immersed in 70% ethanol for 2 min. Subsequently, the sections were transferred into solution B for 10 min, then incubated with FJC working solution C for 10 min at 37°C. Finally, the sections were stained for 5 min with DAPI. Images were obtained with a fluorescence microscope (Nikon).

**Nissl staining**

Nissl staining was used to evaluate neuronal damage. The frozen section preparation process was the same as described above. After hydration for 5 min, brain tissue sections were incubated for 30 min at 37°C with Nissl staining solution (Nissl Stain Solution, G1432, Solarbio, Beijing, China). The slices were dehydrated in gradient alcohol (first 95% alcohol, 3-5s, then 100% alcohol, 1min), then sealed with resin. Images were obtained and analyzed under a light microscope (Leica-DM2500, Wetzlar, Germany).

Cultured cells were collected and resuspended in PBS. Cell viability was tested with LIVE/DEAD Calcein AM-FITC/PI-PE Double Stain Kit (Bestbio, jiangsu, China) or Annexin V-APC/PI-PE Apoptosis Kit (Tenubio, guangzhou, China). Cells do not need to be fixed with 4% formaldehyde. Added 5ul Annexin V or Calcein AM working solution to each 500μl cell suspension and incubated the cells at room temperature for 15 minutes. Added 1ul PI in the cell suspension at the last 5min of incubation. As soon as possible, analyze the stained cells by flow cytometry (Beckman, Shanghai, China).

**TNF-α Secretion of Cerebrospinal Fluid of Mice Assay**

Enzyme-linked immunosorbent assay (ELISA, MM-0132M1, Jiangsu Meimian Industrial Co., Ltd., Jiangsu, China) was uesd to detect the secretion of TNF-α in cerebrospinal fluid of mouse according to the manufacturer’s instructions.

Cerebrospinal fluid is drawn from the foramen magnum. The mouse was fixed to the stereotaxic device through the ear bars and incisor holes after being anesthetized with tribromoethanol. The skin and muscles of the mouse neck and skull surface were isolated to fully expose the foramen magnum. The fine capillary pipette were gently placed into the foramen magnum of the mouse. Approximately 5 to 8 μl of cerebrospinal fluid can be obtained from per mouse. The cerebrospinal fluid with different treatment was collected (50μl) for ELISA assay.

**Statistical analyses**

All statistical analyses in this study were executed utilizing R software (Version 4.0.1). The distinction between two groups was appraised through a two-tailed Student’s T-test. Analyses of variances (ANOVA) with Bonferroni post hoc tests were employed for comparisons across multiple groups. Survival disparities were examined via Kaplan-Meier analysis coupled with the log-rank test. Statistical significance was defined at a threshold of p < 0.05.

**Supplementary Figure legends:**

**Supplementary Figure 1.** Experimental design and animal groups.

**Supplementary Figure 2.** The dosage selection of drug . (A) Concentration of TNF-α in the cerebrospinal fluid of mice in different intervention groups, n=3 per group. (B) Time in central zone of mice treated with different doses of TNF-α in the open field test, n=3 per group. (C) Time in central zone of mice treated with different doses of Infliximab in the open field test, n=3 per group. (D) Time in central zone of mice treated with different doses of Rhodomyrtone in the open field test, n=3 per group. (E) The C3 relative gene expression of primary astrocytes treated with different doses of TNF-α. GAPDH was the loading control for the samples; n = 3 per group. (F) The C3 relative gene expression of TNF-α-activated primary astrocytes treated with different doses of Rhodomyrtone. GAPDH was the loading control for the samples. Data are presented as the means ± SD. P values are analyzed using one-way analysis of variance. n = 3 per group. ^*^P < 0.05, ^**^P < 0.01, ^***^P < 0.001, ns = no significance.

**Supplementary Figure 3.** (A) Frequency cross the central zone of mice with different treatment in the open field test. (B) Double fluorescence staining for GFAP (green) and TNFR1 (red) in the DG, CA1, and CA3 of hippocampal in CUMS groups. Scale bar = 100 μm. (C) TNF-α protein expression of hippocampus in the CUMS group was increased, when compared with the Sham group. GAPDH was the loading control for the samples; n = 3 mice per group. (D) Double fluorescence staining for TNFR1 (green) and C3 (red) in the CA3 of hippocampal with TNF-α treatment. Scale bar = 100 μm. Data are presented as the means ± SD. P values are analyzed using one-way analysis of variance.^*^P < 0.05, ^**^P < 0.01, ^***^P < 0.001, ns = no significance.

**Supplementary Figure 4.** The expressions of TNFR1 and C3 were increased in the hippocampal of chronic unpredictable mild stress (CUMS) mice and was aggravated by TNF-α treatment.  Double fluorescence staining for GFAP (green) and TNFR1 (red) or C3 (red) in the CA3 of hippocampal with TNF-α treatment. Co-staining GFAP and TNFR1 (scale bar = 100 μm); co-staining with GFAP and C3 (scale bar = 50μm).

**Supplementary Figure 5.** The frequency cross the center 25% area of the open field in chronic unpredictable mild stress (CUMS) groups were decreased, when compared with the sham group. Infliximab treatment (A), AAV-TNFR1 transfection (B), and Rho treatment (C) reversed the decreases of crossing numbers in the CUMS group. (D) Representative fluorescent images of primary astrocytes co-stained with GFAP (green) and IBA-1 (red) (scale bar = 50 μm). (H) Effect of Rho treatment on primary astrocytes. Representative dot plots showing the gating strategy of calcein AM, and propidium iodide from the different primary astrocytes groups. Data are presented as the means ± SD. P values are analyzed using one-way analysis of variance.^*^P < 0.05, ^**^P < 0.01, ^***^P < 0.001, ns = no significance.
