# Supplementary figures and images for: TNF-α/TNFR1 activated astrocytes exacerbate depression-like behavior in CUMS mice
Source: Cell Death Discov. 2024 May 6;10:220. doi: 10.1038/s41420-024-01987-4 (PMC11074147; doi:10.1038/s41420-024-01987-4)

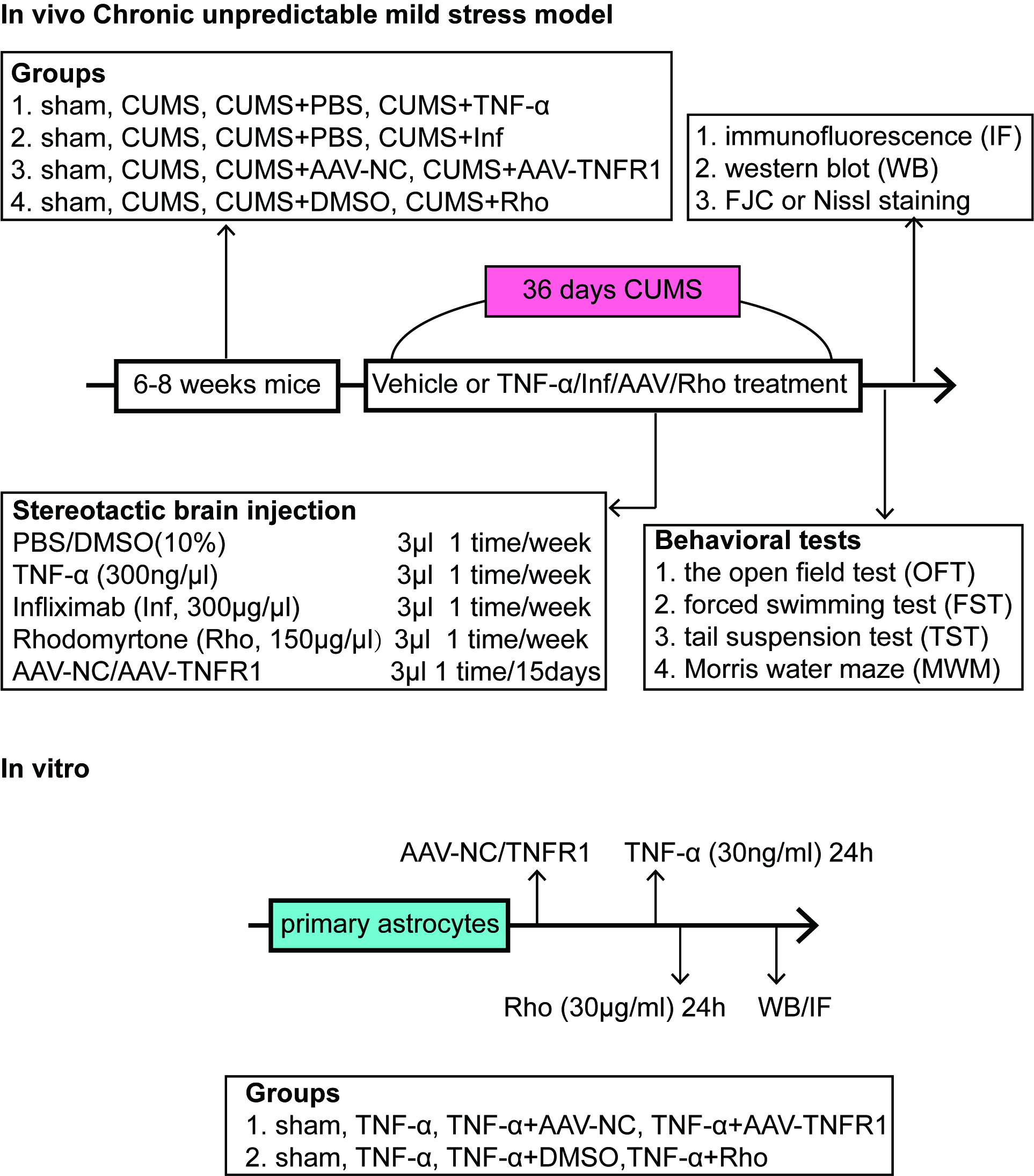

Supplement: Supplementary file 2 — supplemental figure 1 [file 41420_2024_1987_MOESM2_ESM.tif]

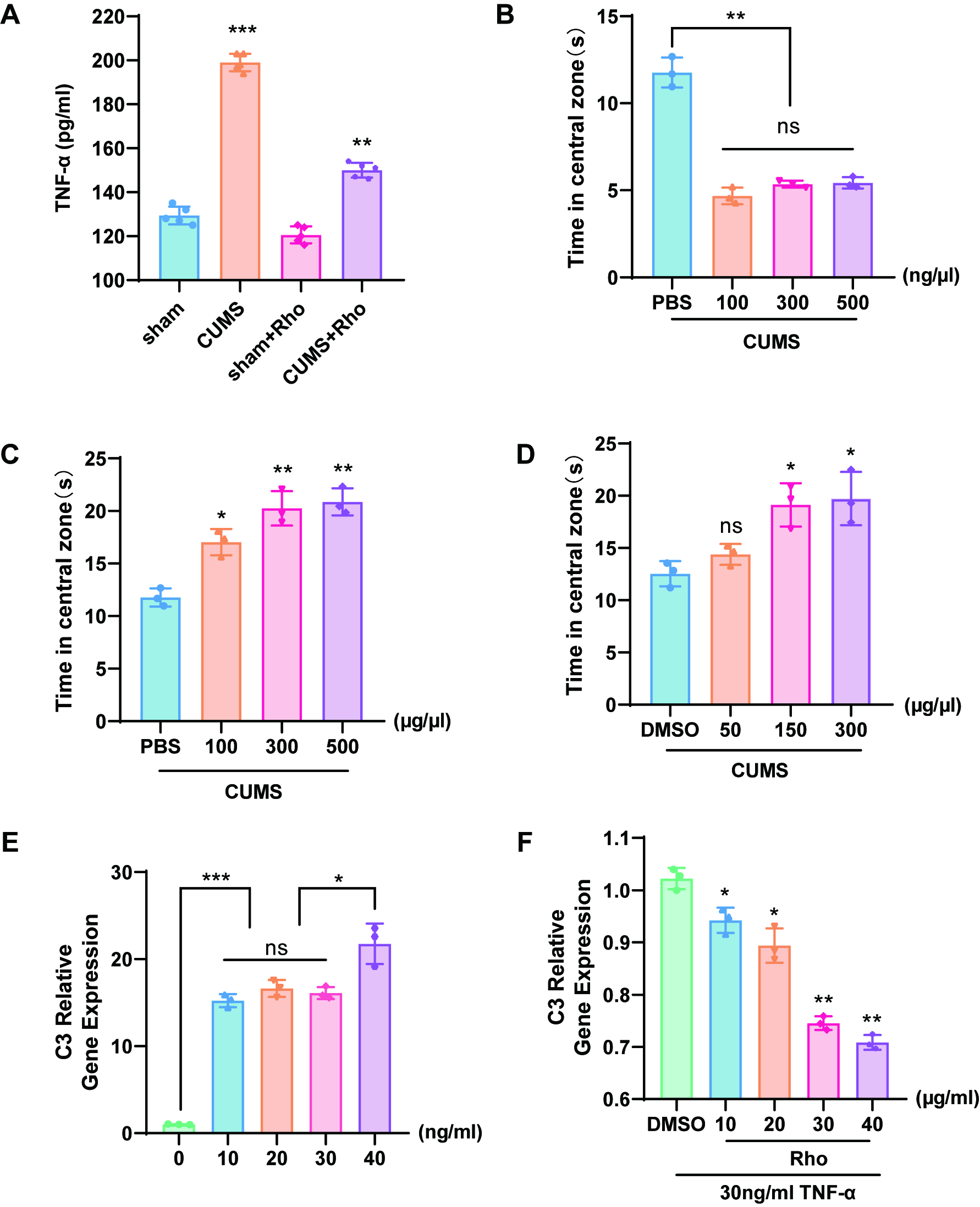

Supplement: Supplementary file 3 — supplemental figure 2 [file 41420_2024_1987_MOESM3_ESM.tif]

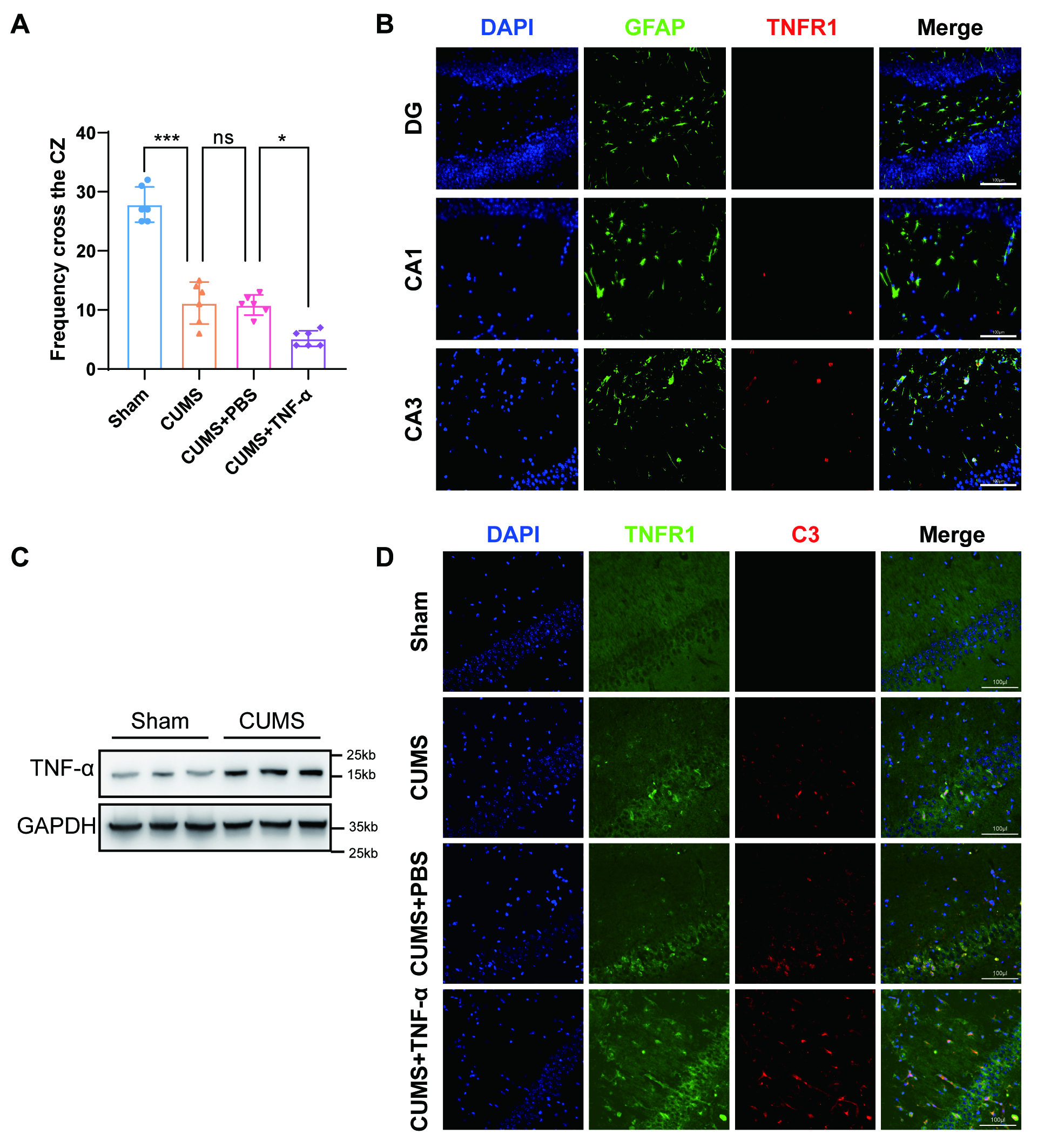

Supplement: Supplementary file 4 — supplemental figure 3 [file 41420_2024_1987_MOESM4_ESM.tif]

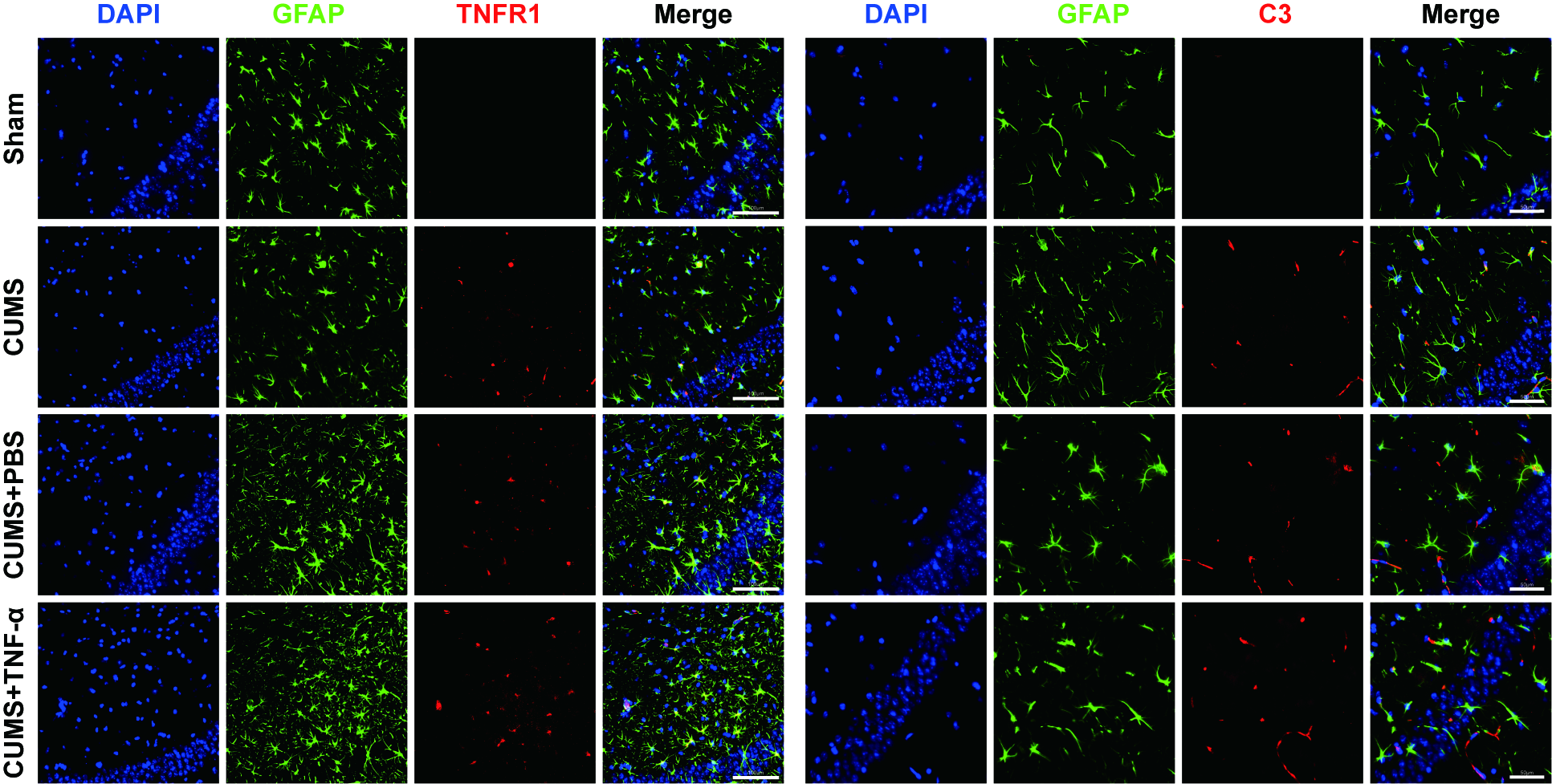

Supplement: Supplementary file 5 — supplemental figure 4 [file 41420_2024_1987_MOESM5_ESM.tif]

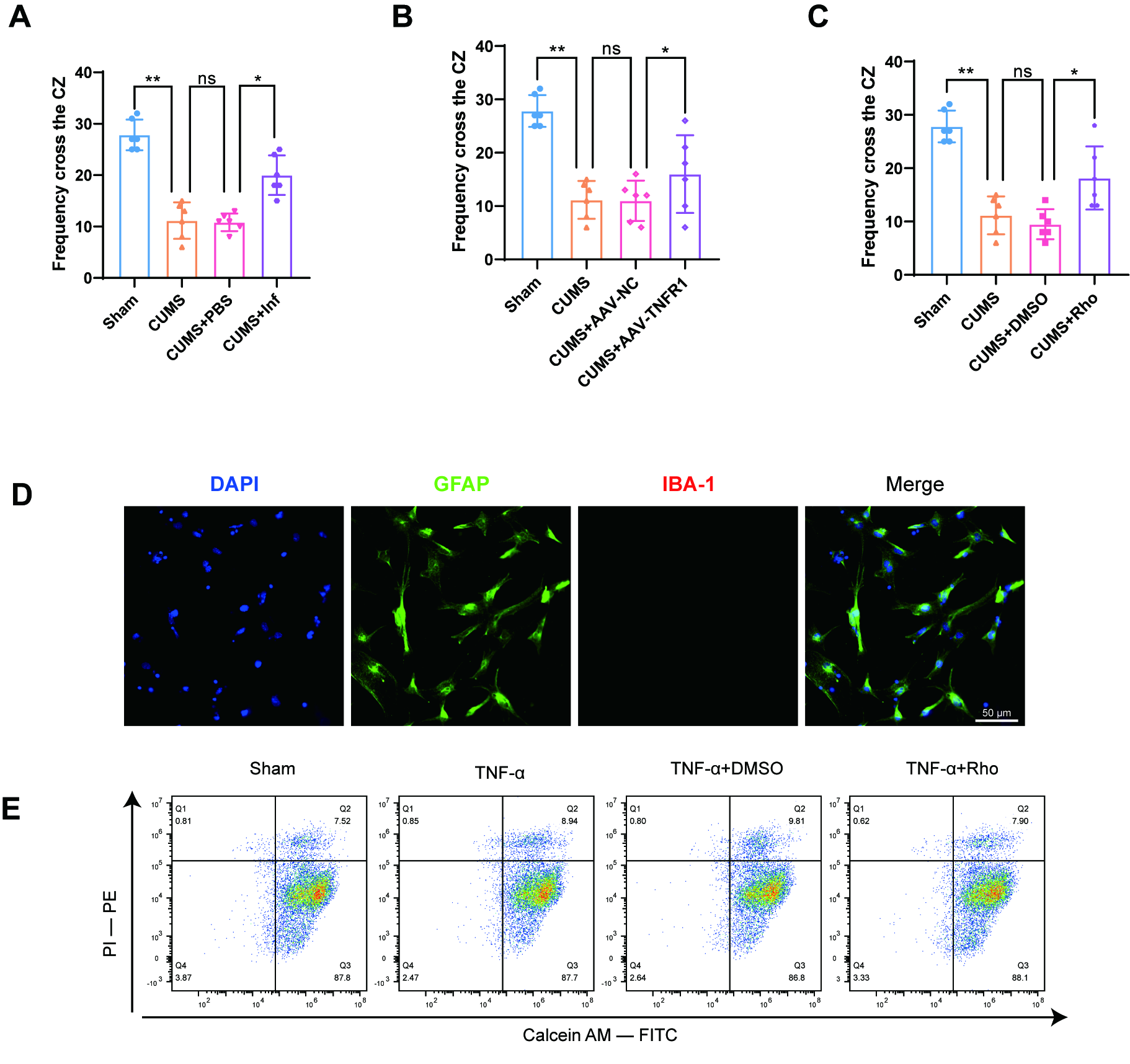

Supplement: Supplementary file 6 — supplemental figure 5 [file 41420_2024_1987_MOESM6_ESM.tif]

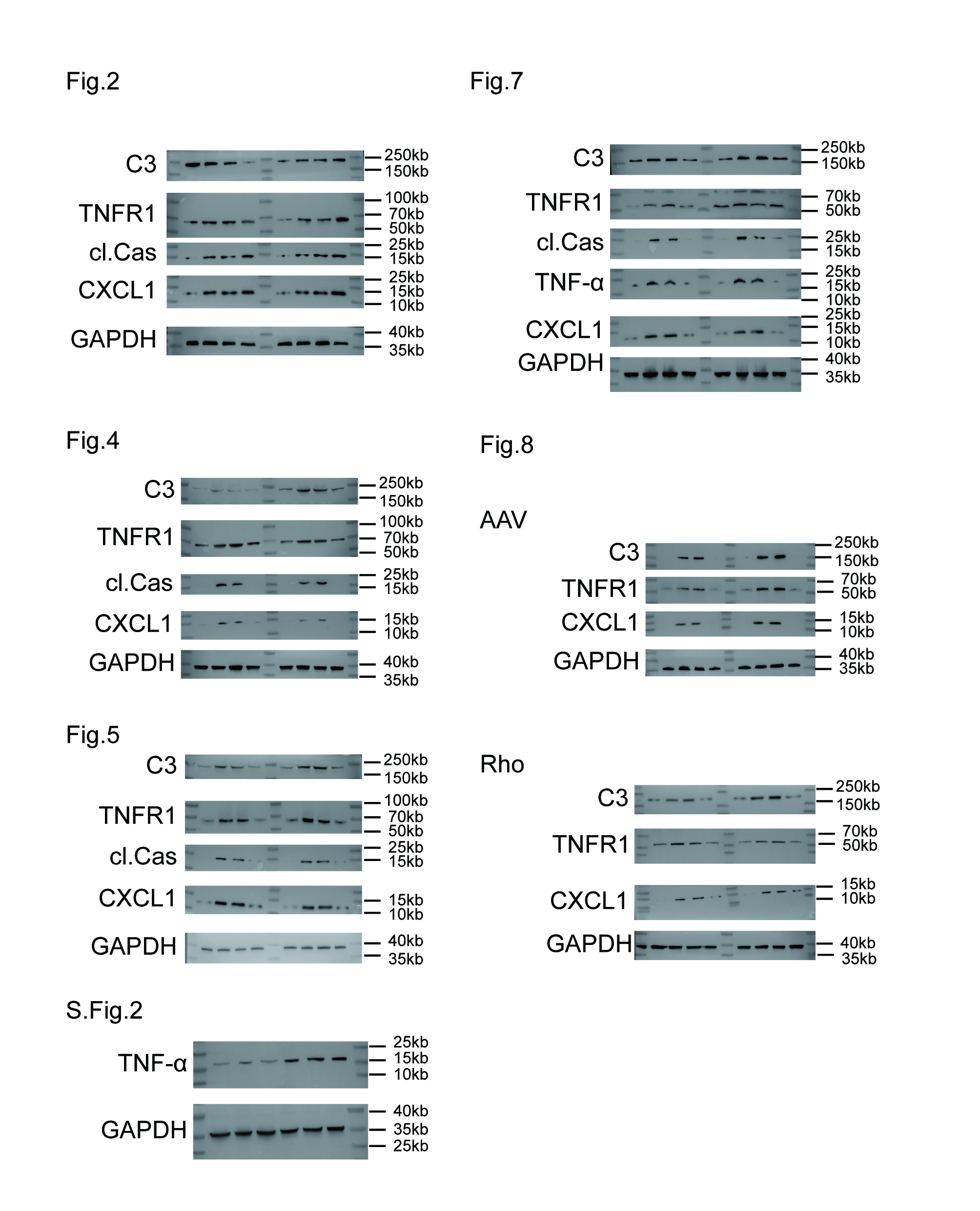

Supplement: Supplementary file 10 — blots [file 41420_2024_1987_MOESM10_ESM.docx]
