# Supplementary material for: TNF-α/TNFR1 activated astrocytes exacerbate depression-like behavior in CUMS mice
Source: Cell Death Discov. 2024 May 6;10:220. doi: 10.1038/s41420-024-01987-4 (PMC11074147; doi:10.1038/s41420-024-01987-4)
Supplement: Supplementary file 7 — Supplementary Table 1 [file 41420_2024_1987_MOESM7_ESM.docx]

**Supplement table 1: Morris water maze start positions.**

| DAY1 | Trail1 | Trail2 | Trail3 | Trail4 |
| --- | --- | --- | --- | --- |
| 1 | N | E | NW | SE |
| 2 | E | NW | SE | N |
| 3 | NW | SE | N | E |
| 4 | SE | N | E | NW |
| 5 | N | NW | E | SE |
| 6 | E | | | |
| Platform: SW | | | | |
