# Supplementary material for: TNF-α/TNFR1 activated astrocytes exacerbate depression-like behavior in CUMS mice
Source: Cell Death Discov. 2024 May 6;10:220. doi: 10.1038/s41420-024-01987-4 (PMC11074147; doi:10.1038/s41420-024-01987-4)
Supplement: Supplementary file 8 — Supplementary Table 2 [file 41420_2024_1987_MOESM8_ESM.docx]

**Supplement Table 2**

AAV9-CMV-TNFR1-EGFP primers used in this study

Primer name Sequence ((5′ → 3′)

| AAV-TNFR1 | Forward | UUGCAAGACAUGUCGGAAATT |
| --- | --- | --- |
|  | Reverse | UUUCCGACAUGUCUUGCAATT |
