# Supplementary material for: TNF-α/TNFR1 activated astrocytes exacerbate depression-like behavior in CUMS mice
Source: Cell Death Discov. 2024 May 6;10:220. doi: 10.1038/s41420-024-01987-4 (PMC11074147; doi:10.1038/s41420-024-01987-4)
Supplement: Supplementary file 9 — Supplementary Table 3 [file 41420_2024_1987_MOESM9_ESM.docx]

**Supplement Table 3**

Real-time PCR primers used for quantification of mRNA expression in this study

Primer name Sequence ((5′ → 3′)

| C3 | Forward | GAGCGAAGAGACCATCGTACT |
| --- | --- | --- |
|  | Reverse | TCTTTAGGAAGTCTTGCACAGTG |
| GAPDH | Forward | AGGTCGGTGTGAACGGATTTG |
|  | Reverse | TGTAGACCATGTAGTTGAGGTCA |
